# Enterococcus hirae Bacteremia Complicated by Septic Spondylitis and Acute Pyelonephritis: A Case Report

**DOI:** 10.7759/cureus.75501

**Published:** 2024-12-10

**Authors:** Tetsufumi Nishi, Yoshihiro Aoki, Kensuke Takahashi, Shuhei Yamano, Koichi Hayakawa

**Affiliations:** 1 Emergency Medicine, Nagasaki Harbor Medical Center, Nagasaki, JPN; 2 Coordination Office for Emergency Medicine and International Response, Acute and Critical Care Center, Nagasaki University Hospital, Nagasaki, JPN

**Keywords:** acute pyelonephritis, bacteremia, enterococcus hirae, sepsis, septic spondylitis

## Abstract

*Enterococcus hirae*, a rare human pathogen, has limited clinical data. This case report presents a case of sepsis, pyelonephritis, and septic spondylitis treated successfully with ampicillin. An 82-year-old woman was hospitalized for acute pyelonephritis and sepsis, presenting with fever and abdominal pain. After admission, her back pain appeared, and magnetic resonance imaging revealed septic spondylitis of the lumbar spine. Blood and urine cultures obtained at admission revealed the presence of *E. hirae*. After six weeks of ampicillin treatment, her symptoms resolved, and she was transferred to a rehabilitation hospital. Retrospectively, a computed tomography scan on admission revealed signs of septic spondylitis, indicating that the patient may have had spondylitis before developing pyelonephritis. *E. hirae*, a rare cause of infection in various organs, responded well to ampicillin. Despite its rarity, spondylitis should be considered in the elderly with low back pain and pyelonephritis for a complete differential diagnosis.

## Introduction

Sepsis remains a leading cause of morbidity and mortality in emergency settings, necessitating rapid and evidence-based interventions for optimal patient outcomes [1]. Among the *Enterococcus* species, *Enterococcus faecalis* and *Enterococcus faecium* are the most common etiologies, accounting for approximately 10% of all human sepsis cases, primarily causing urinary tract and intra-abdominal infections [2]. *Enterococcus hirae*, typically found in birds, cats, and rodents, is exceedingly rare in humans, representing less than 1% of enterococcal infections [3].

The epidemiology and clinical manifestations of* E. hirae* infections have been increasingly documented in recent years, highlighting the emerging importance of this pathogen. A systematic review from 1995 to 2020 identified only 32 cases of human *E. hirae* infections globally, with most presenting as urinary tract infections and biliary tract infections [4]. Subsequent case reports have documented additional rare manifestations including endocarditis [5], osteomyelitis [6], and renal abscess [7]. Furthermore, a recent case series from Switzerland analyzed 14 hospitalized patients with *E. hirae* infections over six years (2016-2022) [8]. The study found that *E. hirae* predominantly affected elderly males (mean age: 68.6 years, 71.4% male) with cardiovascular comorbidities (57.1%). Most infections involved the biliary tract (42.9%) and urinary tract (21.4%). Patients generally responded well to beta-lactam antibiotics, suggesting that *E. hirae* may be a low-virulence pathogen despite its ability to cause diverse clinical infections [8]. The low prevalence but diverse clinical presentations of *E. hirae* infections pose a diagnostic challenge for clinicians. Furthermore, the potential for serious complications and the limited understanding of its pathogenesis underscore the importance of documenting and analyzing each case to better understand risk factors, optimal treatment strategies, and clinical outcomes. The geographical distribution of reported cases suggests that *E. hirae* infections may be underrecognized, particularly in regions with limited microbiological identification capabilities.

Septic spondylitis, particularly relevant in the elderly population, should be considered in the differential diagnosis of fever and back pain in emergency departments, with most cases attributed to *Staphylococcus aureus* and *Streptococcus* species [9]. To contribute to the limited literature on rare infections caused by *E. hirae*, this report presents a case of sepsis caused by *E. hirae*, originating in the southern part of Japan, which led to septic spondylitis and acute pyelonephritis and was successfully treated with ampicillin.

## Case presentation

**Figure 1:**
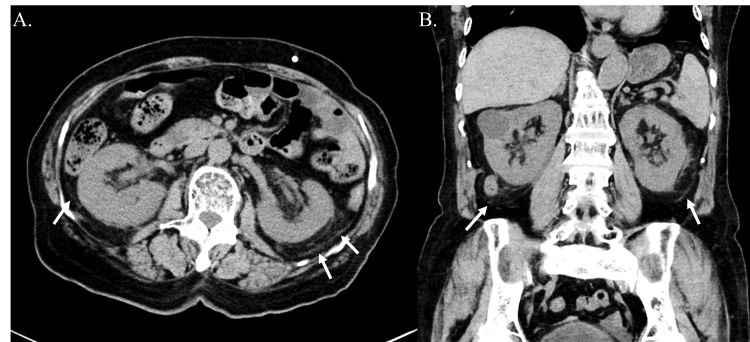
The renal computed tomography upon admission (A) Axial CT scan showing bilateral renal pelvic dilation, thickened renal pelvic walls, and inflammatory changes in perirenal fat appearing as cord-like opacities (arrows). The contrast was adjusted for better visualization. (B) Coronal CT scan confirming the bilateral nature of these findings, with evident renal pelvic wall thickening and surrounding inflammatory stranding in the perirenal fat tissue (arrows). The contrast was adjusted for better visualization.

An 82-year-old woman presented with chief complaints of fever, abdominal pain, and nausea. Living with her husband, she had previously been independent in activities of daily living. Her living environment was clean and well-maintained, with no recent contact with birds or cats. She had a history of myocardial infarction and hypertension and was on aspirin and antihypertensive therapy. However, she had no history of diabetes, chronic kidney disease, or long-term steroid use. Two weeks before her current presentation, she had experienced diarrhea and abdominal pain. The day before hospital admission, the patient reported abdominal pain, general malaise, and mild fever. She was admitted to the emergency department with worsening symptoms. At admission, she had a fever of 39.0°C and required minimal oxygen supplementation. Her blood pressure was 145/79 mmHg, and her pulse was 99 beats per minute. Physical examination revealed tenderness from the left lower abdomen to the midline of the lower abdomen, with no costovertebral angle tenderness or spinal percussion pain. Owing to her malaise, she was unable to walk steadily or stand. Blood tests revealed abnormalities: white blood cell count was elevated at 10,400/µL, platelet count slightly decreased to 118,000/µL, renal function was impaired with creatinine levels at 1.20 mg/dL, and markers of inflammation were high with C-reactive protein (CRP) at 12.71 mg/dL and procalcitonin at 1.55 ng/dL. Blood gas analysis revealed lactic acid levels of 8.0 mg/dL. Urinalysis was negative for nitrites but showed pyuria and bacteriuria. Abdominal computed tomography (CT) revealed funicular opacity in the perinephric fat, mild bilateral renal pelvic dilatation, and thickening of the renal pelvic walls (Figure 1, Panels A and B).

**Figure 2:**
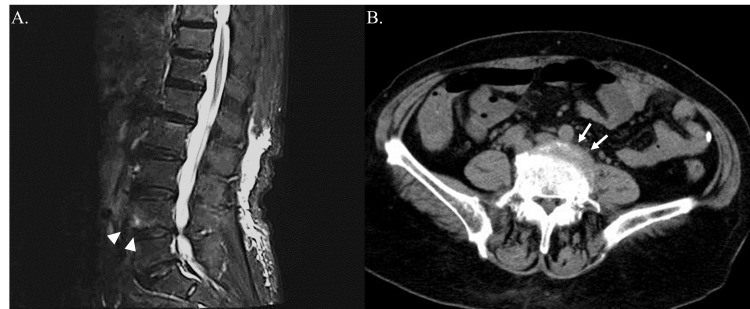
The magnetic resonance imaging and the computed tomography of the lumbar spine (A) Sagittal view of the magnetic resonance imaging of the lumbar spine on the third day of hospitalization. The short tau inversion recovery (STIR) sequence revealed high signal intensity areas near L4/5 (arrowheads), in addition to preexisting spinal canal stenosis. The contrast was adjusted for better visualization. (B) Axial view of the computed tomography of the lumbar spine on admission. Mild opacity in the fatty tissue around the L4-5 vertebral body was observed (arrows).

The patient was diagnosed with sepsis based on suspected infection and an acute increase in Sequential Organ Failure Assessment (SOFA) score of two points (creatinine: 1.2-1.9 mg/dL, platelet count < 150,000/μL), meeting sepsis-3 criteria, and acute pyelonephritis without urinary tract obstruction based on urinalysis and imaging findings. Due to the high prevalence of ESBL-producing *Escherichia coli*, treatment with cefmetazole (CMZ) was initiated. The following day, the fever persisted, reaching up to 40°C, and blood cultures identified Gram-positive cocci in chains. While antimicrobial susceptibility testing was still pending, vancomycin was empirically administered given the possibility of *Enterococcus* infection. On the third day of hospitalization, magnetic resonance imaging (MRI) of the lumbar spine was performed because of lower back pain, revealing high signal intensity near L4/5 on short tau inversion recovery (STIR) images and lumbar spinal canal stenosis, but no epidural abscess (Figure 2, Panel A). Retrospectively, mild opacity was also observed in the fatty tissue surrounding L4-5 vertebral bodies on the CT scan taken at admission (Figure 2, Panel B). This led to a diagnosis of septic spondylitis.

Ultimately, *E. hirae* was identified in both blood and urine cultures. Colony characteristics, negative catalase test, and positive pyrrolidonyl aminopeptidase (PYR) test were used to identify the isolate as *Enterococcus* species. The organism formed small, gray, non-hemolytic colonies on blood agar plates. A negative catalase test was confirmed by the absence of bubble formation when colonies were mixed with 3% hydrogen peroxide, and a positive PYR test was demonstrated by the development of a red color when colonies were tested with PYR substrate. Further species identification as *E. hirae* was performed using the BD Phoenix 100 system (Becton Dickinson, Franklin Lakes, NJ), an automated microbial identification system that utilizes biochemical and enzymatic test reactions to identify bacterial species. On the fourth day of admission, the antibiotic regimen was changed to ampicillin based on drug susceptibility testing results (Table 1). Echocardiography and head MRI revealed no signs of infective endocarditis or cerebral embolism. A final diagnosis of *E. hirae* infection, including bacteremia, acute pyelonephritis, and septic spondylitis, was made. With the administration of ampicillin, the patient’s fever subsided, and her inflammatory markers improved. By the seventh day of her hospital stay, she was able to mobilize with the aid of a corset. After four weeks, the patient was transferred to another rehabilitation facility. Following six weeks of intravenous antibiotic treatment, a follow-up at two months revealed a significant improvement in her condition. She regained independence in daily activities, and her lower back pain resolved.

**Table 1:** Drug susceptibility testing results CLDM: Clindamycin; ABPC: Ampicillin; PIPC: Piperacillin; PCG: Penicillin G; MINO: Minocycline; VCM: Vancomycin; TEIC: Teicoplanin; DAP: Daptomycin; LZD: Linezolid; ST: Sulfamethoxazole/Trimethoprim; MUH: Multi-use hypochlorite; GMH: Gentamicin (high-dose); CEZ: Cefazolin; CFX: Cefuroxime; IPM/CS: Imipenem/Cilastatin; A/S: Ampicillin/Sulbactam; GM: Gentamicin; AMK: Amikacin; ABK: Arbekacin; EM: Erythromycin.

Drug Name	MIC	Susceptibility
CLDM	>2	R
ABPC	≤1	S
PIPC	>2	R
PCG	≤2	S
MINO	≤1	S
VCM	≤0.5	S
TEIC	≤0.5	S
DAP	4	S
LZD	2	S
ST	≤1/19	R
MUH	≤256	
GMH	≤500	S
CEZ	>16	R
CFX	>8	R
IPM/CS	≤2	
A/S	≤4/2	
GM	8	R
AMK	32	R
ABK	8	R
EM	≤0.25	S

## Discussion

The report described a case of sepsis caused by *E. hirae*, a rare *Enterococcus* species, which presented with urinary tract infection and septic spondylitis. The patient responded well to antimicrobial therapy with ampicillin and had a favorable outcome. At admission, CT scans revealed indications of septic spondylitis, which suggested that the spondylitis either occurred concurrently with or preceded the urinary tract infection. However, the exact mechanisms underlying this complication remain unclear.

In a retrospective study from Nagasaki University Hospital [10], risks for *Enterococcus* infection were generally reported to include abnormal urinary tract structure, hypoalbuminemia, immunosuppressive medications, and device implantation; however, the specific risks for *E. hirae* are unknown. A recent literature review on 32 cases of *E. hirae* infections reported that more cases are complicated by kidney disease, cirrhosis, and diabetes mellitus [4], although none were observed in the current case. The median age of the patients was 63 years, with a higher prevalence in men (64.5%). Infections caused by *E. hirae* can be relatively common in the elderly but also occur in younger people. A case report by Bollam et al. described a 33-year-old man with no significant past medical history who developed right tibial osteomyelitis due to *E. hirae* after trauma [6]. In addition, a 21-year-old woman with type 1 diabetes mellitus was reported to have kidney abscess and bacteremia due to *E. hirae *[7].

The drug susceptibility of *E. hirae* is generally favorable. A 2021 report noted four cases of gentamicin resistance [4]. In most cases, treatment with penicillins such as ampicillin, cephems like cefmetazole and cefazolin, and vancomycin was effective [4]. However, in the present case, cefmetazole was not included in our drug susceptibility testing panel and did not improve symptoms. Treatment with ampicillin, however, proved successful.

The reported common infections by *E. hirae* include urinary tract, biliary tract infections, and infective endocarditis [4,5,8]. The review also reported a case of septic spondylitis [4]. However, according to the literature review and subsequent published cases since 2021, there have been no reported instances of concurrent acute pyelonephritis and septic spondylitis caused by *E. hirae*. In general, few cases of concurrent acute pyelonephritis and septic spondylitis have been reported. To understand the mechanism underlying the complications of urinary tract infection and spondylitis, past case reports have indicated several pathways besides complications from septic embolism caused by infective endocarditis. In a case documented by Takeuchi et al. in 2012, the patient experienced repeated urinary tract infections, and blood cultures revealed *E. faecium *[11]. They discussed the potential mechanism of the concurrent condition, suggesting that retrograde infection through the paraspinal venous plexus from pyelonephritis was the most likely cause of septic spondylitis. Another possible mechanism for the complication is hematogenous spread, as suggested in a case report [12]. In the report, the patient had been treated with antibiotics by a previous physician, and various cultures were negative; however, the patient had a history of periodic arthrocentesis of the right knee, which led to right knee arthritis and subsequently resulted in a multi-organ infection. In our present case, CT scans showed signs of spondylitis at the time of the patient’s admission and onset of pyelonephritis, with infective endocarditis being ruled out, at least by transthoracic echocardiography. The patient's initial onset of spondylitis was probably due to asymptomatic transient bacteremia secondary to previous enteritis. When dealing with cases of fever and back pain in the elderly, it is crucial to meticulously exclude other differential diagnoses based on the progression of the disease rather than hastily concluding a diagnosis of a single disease, such as acute pyelonephritis.

## Conclusions

This case adds to the medical literature as the first reported instance of concurrent acute pyelonephritis and septic spondylitis caused by *E. hirae*, expanding our understanding of this pathogen's potential clinical manifestations. While *E. hirae* demonstrates similar drug susceptibility to *E. faecalis* and responds well to ampicillin treatment, this case highlights the importance of considering atypical presentations and multiple organ involvement, even with rare pathogens. The successful treatment outcome underscores that thorough diagnostic evaluation of elderly patients with complex infections should include careful assessment for concurrent infections at anatomically distinct sites, as this may impact the duration and approach to antimicrobial therapy.
